# The promise of digital herbarium specimens in large‐scale phenology research

**DOI:** 10.1111/nph.70178

**Published:** 2025-05-19

**Authors:** Natalie Iwanycki Ahlstrand, Richard B. Primack, Matthew W. Austin, Zoe A. Panchen, Christine Römermann, Abraham J. Miller‐Rushing

**Affiliations:** ^1^ Natural History Museum of Denmark University of Copenhagen Copenhagen 1350 Denmark; ^2^ Boston University Boston MA 02215 USA; ^3^ Herbarium Missouri Botanical Garden St Louis MO 63110 USA; ^4^ Department of Biology Acadia University Wolfville NS B4P 2R6 Canada; ^5^ Plant Biodiversity Friedrich Schiller University Jena Jena 07743 Germany; ^6^ Senckenberg Institute for Plant Form and Function (SIP) Jena 07743 Germany; ^7^ German Centre for Integrative Biodiversity Research (iDiv) Halle‐ Jena‐ Leipzig Leipzig 04103 Germany; ^8^ US National Park Service Acadia National Park Bar Harbour ME 04609 USA

**Keywords:** climate change, digitization, herbarium specimen, international collaboration, plant phenology, spatiotemporal scale

## Abstract

The online mobilization of herbaria has made tens of millions of specimens digitally available, revolutionizing investigations of phenology and plant responses to climate change. We identify two main themes associated with this growing body of research and highlight a selection of recent publications exemplifying: investigating phenology at large spatial and temporal scales and in understudied locations and testing long‐standing theories and novel questions in ecology and evolution that were not previously answerable. We explore strengths and limitations of using digitized herbarium specimens in phenology research, including: issues of sampling; reliability, transferability, and biases; and ethical and social justice considerations. This field will see further breakthroughs as herbaria around the world continue to mobilize and digitally interlink their collections. New developments will likely come from advances in technology, international collaborations, and including understudied plant taxa and regions such as the Arctic and the tropics. Advances in technology are already improving digitization workflows and speeding the collection of phenology data from digital specimens.

## Disclaimer

The New Phytologist Foundation remains neutral with regard to jurisdictional claims in maps and in any institutional affiliations.

## Digitization of specimens opens opportunities

**Fig. 1:**
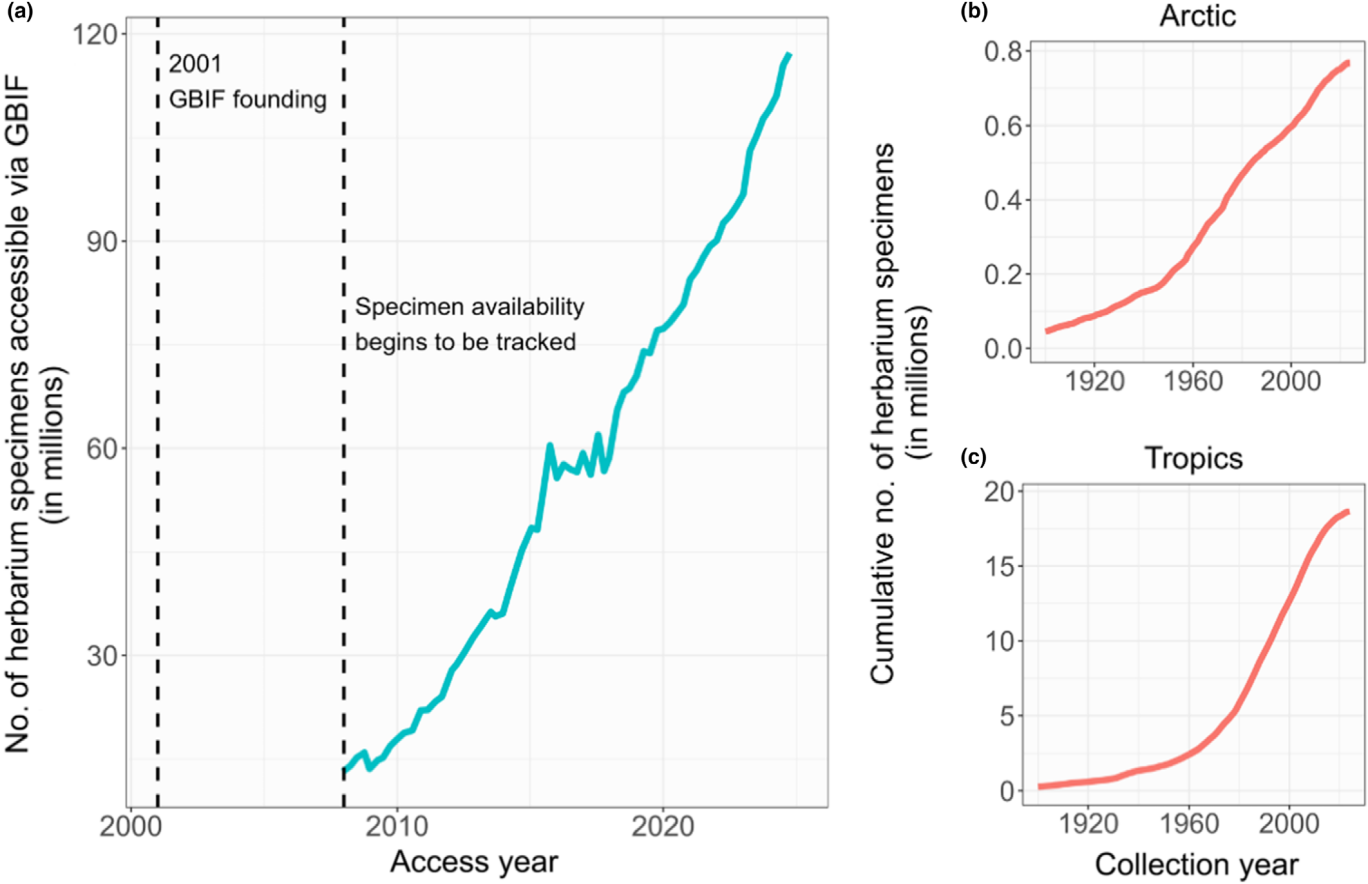
Increase in herbarium specimen availability through time. (a) The cumulative number of herbarium specimen occurrences accessible via the Global Biodiversity Information Facility (GBIF) over time from when availability tracking began. The cumulative number of herbarium specimens per collection year from (b) the Arctic and (c) the tropics. Specimen numbers in panels (b) and (c) derive from a GBIF occurrence search for ‘Basis of Record’ = ‘Preserved Specimen’, ‘Occurrence status’ = ‘present’, and ‘Scientific name’ = ‘Plantae (kingdom)’ (GBIF, 2024). Panels (b) and (c) only include counts of specimens with digitized coordinates; tropical specimens defined as those occurring between 23.5°S and 23.5°N, Arctic specimens defined as those occurring above 66.57°N.

Herbarium specimens provide unique sources of data that have proved invaluable to researchers addressing questions in climate change biology, ecology, and evolution at wider taxonomic, phylogenetic, geographic, and temporal scales than almost any other source of biodiversity data (Willis *et al*., 2017; Davis, 2023; Eckert *et al*., 2024). Specimen digitization efforts world‐wide and the simultaneous development of online portals that facilitate searching and filtering through digital specimen data have dramatically improved accessibility to herbarium collections (Lang *et al*., 2019; Davis, 2023). Instead of physically traveling to herbaria or relying on cumbersome and slow mailing of specimens, researchers can now access millions of digital herbarium specimen records and images from anywhere with internet access. Large‐scale digitization efforts have been coordinated at regional and national levels by consortia such as the Integrated Digital Biocollections (iDigBio), the Distributed System of Scientific Collections (DiSSCo), and the Australasian Virtual Herbarium and at the global level by the Global Biodiversity Information Facility (GBIF). The number of digital specimens accessible through GBIF has been steadily increasing at a rate of *c*. 6.1 million specimen records yr^–1^ (> 118 million preserved plant specimen records available on GBIF as of 2024, including > 50 million with images; GBIF, 2024) (Fig. 1a).

Several recently published papers using thousands of digital specimens demonstrate the benefits of digitization campaigns across herbaria. For example, researchers have gained insights into plant phenology across broad spatiotemporal scales and have tested theories and addressed novel questions in ecology and evolution that were not previously answerable (Davis, 2023). Moreover, these long‐term, time series data capture more climatic variation than more limited time spans (Iwanycki Ahlstrand *et al*., 2022) and can span regional and even continental spatial scales that allow for the detection of phenology patterns not found in other more localized plant data (Ramirez‐Parada *et al*., 2024).

At the same time, we need to be aware of biases and other sources of error associated with digitized herbarium specimens, which might lead researchers to reach erroneous conclusions (Daru *et al*., 2018; Panchen *et al*., 2019), and ethical and social justice considerations, so we can avoid compounding past harms associated with the colonial history of plant collections (Park *et al*., 2023c).

In this viewpoint article, we describe how large numbers of digitized herbarium specimens have recently been leveraged to address questions in phenology and climate change research. We discuss two main themes that have emerged from this growing body of research and highlight a selection of recent papers exemplifying them: (1) investigating phenology at large spatiotemporal scales and in understudied locations and (2) testing long‐standing theories and novel questions in ecology and evolution. We then explore the strengths and limitations of using digital herbarium specimens in ecological and evolutionary research, and the future directions suggested by these recent papers.

## Scaling up from local to cross‐continental and understudied regions of the world

### Cross‐continental scales

**Fig. 2:**
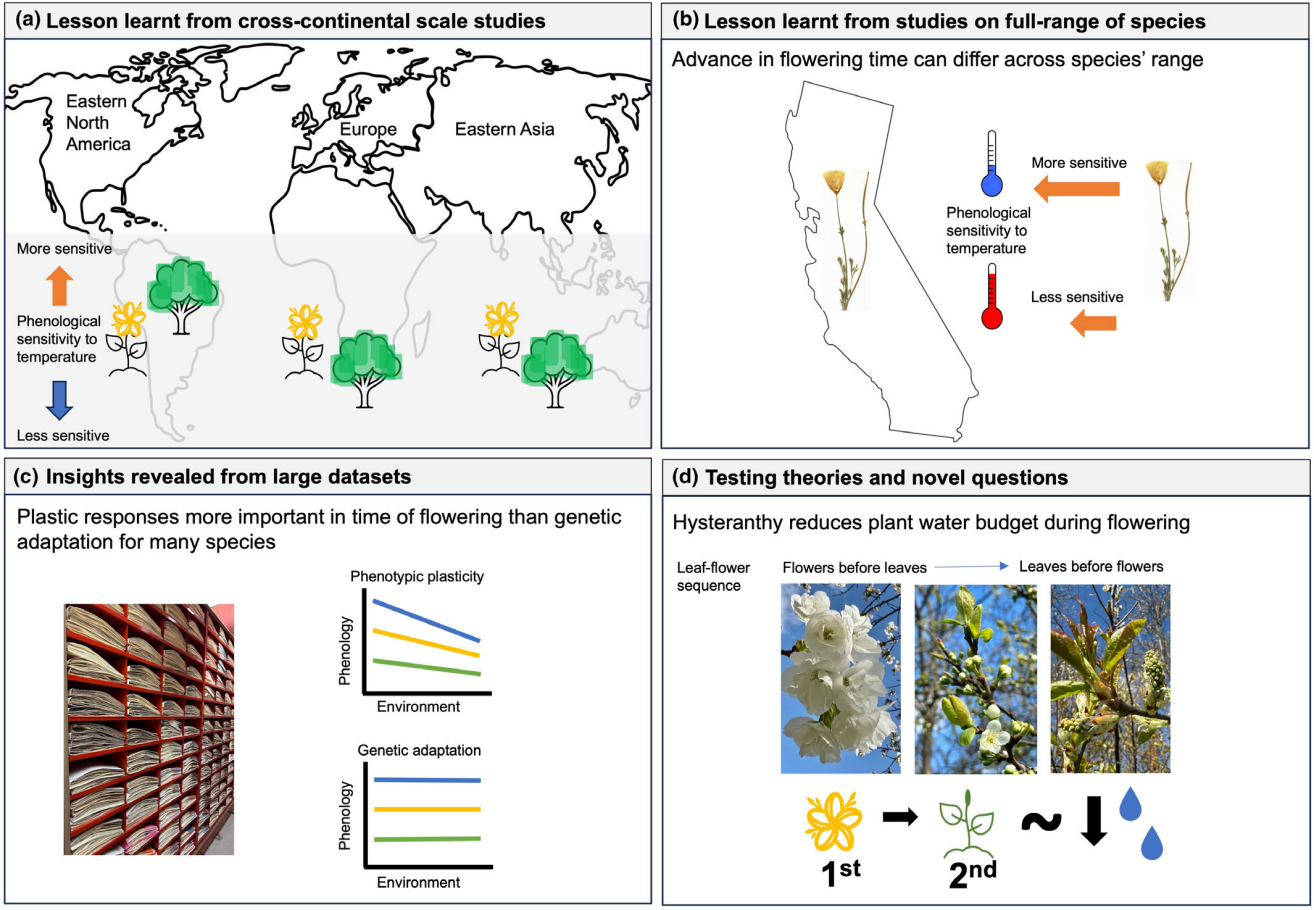
Overview of recent studies revealing various approaches relying on large phenology data sets from herbarium specimens. (a) Lee *et al*. (2022) used specimens from North America, Europe, and Asia to demonstrate cross‐continental differences in the degree of phenological escape; (b) Pearson *et al*. (2021) used thousands of specimens spanning the full range of the California poppy (*Eschscholzia californica* Cham.) to show this species' flowering responds differently to climate across its range; (c) Ramirez‐Parada *et al*. (2024) used over a million specimens to test whether plastic responses are more important in explaining phenology than adaptation; (d) Buonaiuto *et al*. (2024) scored flower‐leaf sequences using thousands of specimens in American plum species (*Prunus* L. subgen. *Prunus* sect. *prunocerasus*) showing that the degree of flowering before leaf‐out positively correlates with the degree of drought stress.

Online access to millions of specimens collected world‐wide gives researchers the potential to scale up and test whether phenological patterns observed at local scales can be seen at the continental and even cross‐continental scales. For example, a detailed study of phenology in one location in Concord, MA, USA, building on the observations of the environmental philosopher Henry David Thoreau, found that leaf out times of tree species were more sensitive to temperature than flowering times of herbaceous wildflower species, leading to the possibility of a reduced window for phenological escape (Heberling *et al*., 2019). To determine whether this pattern also occurred at larger spatial scales, Lee *et al*. (2022) used 5522 herbarium specimens, accessed and evaluated by an international team, to compare the phenology of trees and wildflowers in eastern North America, Europe, and East Asia. Differences in the climate sensitivity of leaf out of trees and flowering times of wildflowers were found to be greatest across eastern North America, leading to the possibility that the phenomenon observed in Concord also occurred across much of the eastern portion of the continent. However, there was no apparent difference between the climate sensitivity of tree and wildflower phenology in East Asia and Europe (Fig. 2a; Lee *et al*., 2022), illustrating that phenological patterns are not always consistent across continents. This study illustrates the tremendous opportunities provided by digitized herbarium studies to investigate phenological patterns over vast spatial scales.

### Full ranges of species

Digitized herbarium specimens can also allow researchers to investigate spatial patterns in phenological sensitivity to environmental variables across the full ranges of species, something not previously possible. For example, a study using 393 specimens of California poppy (*Eschscholzia californica* Cham.) across its range showed that flowering times were more sensitive to temperature in cooler parts of its range, and advanced more in response to warming in these regions (Pearson *et al*., 2021, Fig. 2b). Miller *et al*. (2023) used 3797 herbarium specimens of ten tree and seven wildflower species across their ranges in eastern North America and found that native trees and wildflowers were more sensitive to temperature in the warmer, southern parts of their range. Panchen & Gorelick (2017) also revealed spatial variation in temperature sensitivity in a study using 3795 specimens, which found that flowering times of 23 Arctic species were more sensitive in the northern and eastern parts of their range. These types of studies using herbarium specimens demonstrate that species may be responding to changes in climate heterogeneously across the species' range.

### Understudied locations

Digitally available specimens allow researchers to carry out phenological research in understudied areas of the world, such as the tropics (Lima *et al*., 2021; Davis *et al*., 2022; Park *et al*., 2023a) and the Arctic (Panchen & Gorelick, 2017; Panchen *et al*., 2019; Iwanycki Ahlstrand, 2023a). Despite high and increasing specimen availability (Fig. 1c), the use of herbarium specimens in the study of tropical phenology is still in its infancy. A recent study by Park *et al*. (2023a) may help reverse this trend by demonstrating that phenological data from 4638 herbarium specimens closely match phenology from field studies for 26 tropical species. In the Arctic – an area with comparatively fewer herbarium specimens and species (Fig. 1b) – studies are further revealing the value of digital specimens for phenological research. In a study of 17 000 digital herbarium specimen images, Z.A. Panchen, J.M. Saarela, J. Doubt, and H.M. Kharouba (unpublished data) found that the flowering times of 97 Arctic species are converging; that is a contraction in the flowering season due to the advancement of flowering in late flowering species but not early flowering species, which concurs with a synthesis of Arctic and alpine phenology field monitoring data (Prevéy *et al*., 2019). In summary, these studies illustrate the value of digitized specimens to provide insight into the effects of climate change on phenology in places of the world where field studies are logistically challenging to conduct.

## Testing theories and novel questions

### Genetic adaptation vs phenotypic plasticity

With tens of millions of specimens now available online (GBIF, 2024), plant scientists are realizing the potential for using digital herbarium specimens to test key ideas of climate change science and evolutionary ecology. As an example, consider that for over a century, evolutionary ecologists have investigated the relative contributions of genetic adaptation vs phenotypic plasticity in determining phenotypic expression in local site conditions. They have commonly studied this topic through common garden experiments, reciprocal transplant experiments, and genetic studies. In a new paper, Ramirez‐Parada *et al*. (2024) measured flowering‐time sensitivity over time and space using 1038 027 herbarium specimens of 1605 species across a variety of North American ecoregions. The authors used temporal sensitivity as a proxy for plasticity and spatial sensitivities as a proxy for adaptation and plasticity to demonstrate that for most species, plastic responses were more important than genetic adaptation for explaining flowering time variation across temperature gradients (Fig. 2c). Moreover, the importance of plasticity varied by ecological region. Multi‐generational data sets are needed to infer genetic adaptation. These analyses required phenology data from specimens spanning > 120 yr and would not have been possible without access to millions of digital herbarium specimens from multiple collections. Phenology data gathered at one or just a few locations or covering only short periods of time would not have revealed this pattern.

### Leaf‐flower sequencing

In another example, digital herbarium specimens were used to investigate hysteranthy, which is a striking but not well‐understood phenomenon in which some species of trees and shrubs flower before they leaf out. Buonaiuto *et al*. (2024) used 2521 specimens of nine American plum species (*Prunus* L. subgen. *Prunus* sect. *prunocerasus*) and developed a new approach to quantify flower–leaf sequence variation. The research team tested two hypotheses that have long been proposed, but rarely tested due to insufficient data: that hysteranthy may promote increased tolerance to aridity and/or greater visibility to pollinators. They modelled the relationships of flower‐leaf sequence determined from specimens and petal length measured from specimens, and a standardized drought tolerance index for each species. The main conclusion was that the degree of hysteranthy positively correlates with the degree of drought stress, suggesting that hysteranthy might serve to reduce a plant's water budget during the flowering period (Fig. 2d).

### Sex differences in response to climate change

A novel question that digital specimens have been used to address is whether there are intraspecific sex differences in phenological responses to climate change. Differences in the secondary sex expression of plants are well known, with male plants often producing larger and more flowers than female plants. Xie *et al*. (2023) studied differences in the sex expression of flowering phenology of nine cottonwood species (*Populus* L.) using 13 761 digitized herbarium specimens. They found that male flowering is generally earlier and more sensitive to temperature than female flowering, suggesting that there may be reduced cross‐pollination between male and female plants, and lower seed set in these species, as the climate continues to warm. Such an unexpected result needs to be examined further in field investigations and experiments.

### Effects of urbanization

While it is well known that urban areas are warmer than neighbouring rural areas with earlier flowering and green‐up (Neil *et al*., 2010), Park *et al*. (2023b) used over 70 000 specimens representing 200 species from the eastern United States to reveal that the effects of urbanization on the timing of flowering and fruiting vary across species and space. Increased urbanization results in earlier flowering in cooler and wetter regions and delayed fruiting in regions with wetter springs. Impacts along an urbanization gradient are often overlooked in studies using herbarium specimens, but this study suggests that future changes in phenology and their impacts on ecosystems should consider urbanization.

### Informing grass pollen allergy season

Digital specimens have been used to develop a new way to investigate the relative contributions of different grass species to pollen allergies (Iwanycki Ahlstrand *et al*., 2023b). Because grass pollen looks morphologically similar across species, pollen monitoring schemes are unable to pinpoint which grass species are causing people's allergies. Flowering times for 12 allergenic pollen producing grass species were determined using 3854 digital specimens collected in Denmark. Seven grass species' flowering times overlapped with the times when grass pollen in the atmosphere was at its peak, and the flowering times of four species were found to be particularly sensitive to climate warming and are likely driving the earlier and longer pollen seasons for allergy sufferers. These four grass species can then be made the focus of further monitoring efforts and the target of management efforts to reduce pollen levels.

## Strengths and limitations of digital specimens as sources of phenological data

As studies using large numbers of digitized herbarium specimens become available, questions arise about sampling bias and data reliability, as well as ethical and social justice considerations. In this section, we describe these questions so researchers can be aware of how they might influence their own work. We suggest ways that researchers can address many of these issues, but in most cases, solutions will vary depending on specific research projects.

### Sampling digital specimen data

Large‐scale phenology studies can require searching through and obtaining data from multiple digital specimen repositories (e.g. Lee *et al*., 2022; Ramirez‐Parada *et al*., 2024). A growing number of scripts and semiautomated tools are available to assist with the download and processing of digital specimen images and associated label data (e.g. Chamberlain & Boettiger, 2017) as well as for harmonizing taxonomy and nomenclature (e.g. Kindt, 2020; Boyle *et al*., 2021). To evaluate the phenological stages of specimens such as flowering or fruiting stages, researchers often must visually inspect them, which can be done at large scales via crowdsourcing or citizen science groups (Willis *et al*., 2017). Machine learning models are also being developed and evaluated for their ability to automatically quantify leaf (Weaver *et al*., 2020) and flower (Davis *et al*., 2020) traits from herbarium specimens, and test for trait–climate associations (Love *et al*., 2021; Wilde *et al*., 2023).

These approaches to gathering and working with large specimen data sets are still in their infancy. Methods are changing, and researchers are still learning how to best handle errors, uncertainties, and biases that are present in specimen data, such as tendencies to collect specimens near roads and urban areas and to provide vague location data (Pearson *et al*., 2020). For those who do not want to wait for crowdsourced or machine learning approaches to mature, researchers can assemble small, well‐trained teams to evaluate fewer specimens, creating smaller data sets with higher quality data (Lee *et al*., 2022; Iwanycki Ahlstrand *et al*., 2023b; Kühn *et al*., 2025).

### Reliability and transferability

It is well documented that phenological data extracted from herbarium specimens covering a wide range of plant taxa at local scales can often provide comparable information to field‐based phenology surveys in temperate regions (e.g. Davis *et al*., 2015; Austin *et al*., 2024). Using simulations, Park *et al*. (2024) found that herbarium data accurately predicted the timing and duration of flowering phenology at the population level. Similarly, Iwanycki Ahlstrand *et al*. (2022) found that the phenological sensitivity of species calculated using herbarium specimens was comparable to that calculated using citizen science data obtained from iNaturalist, and these two data types could be combined to increase sample size and expand the temporal scale (also see Panchen *et al*., 2012; Lee *et al*., 2024). Furthermore, by comparing herbarium phenology data to that obtained from the US National Phenology Network, Ramirez‐Parada *et al*. (2022) found that herbarium specimens provided reliable estimates of phenological responses over broad spatial and temporal scales and even estimated taxonomic differences in phenology comparably to field data. However, as we describe below, there can be limitations to the reliability of herbarium specimens in phenology research.

### Biases associated with herbarium specimens

Herbarium specimens are well recognized to have biases that could interfere with phenology research, including taxonomic biases and biases associated with how, where, and when specimens were collected and processed (Daru *et al*., 2018; Panchen *et al*., 2019). For example, if collectors tended to target male plants early in the season because of their larger and more abundant flowers, it might appear that male plants flower earlier than female plants (Xie *et al*., 2023; Yang *et al*., 2023). Similarly, spatial biases such as greater sampling effort close to highly populated areas, which are often warming faster than nearby rural areas, can also skew phenological data, and some taxonomic groups and geographic regions tend to be over‐ or underrepresented in herbaria (Daru *et al*., 2018; Panchen *et al*., 2019). Further, some plant groups are not as amenable as others with respect to determining phenology stages, introducing species or region‐specific biases into herbarium data. We suggest that researchers carefully review data from specimens – particularly for spatial, temporal, and taxonomic spread, dates of collection, and the identity of the collectors – to screen for and address any biases across time, space, or species, as proposed in Panchen *et al*. (2019). Researchers new to analysing herbarium specimen records might consider seeking help from researchers with experience collecting herbarium specimens.

### Biases and errors associated with the digitization process

Digital herbarium collections can also be biased by the digitization process itself. Despite the availability of tens of millions of specimens online (GBIF, 2024; Fig. 1a), most herbaria – including smaller, regional herbaria and even those of many larger institutions – are not yet fully or even partially databased and imaged, rendering them invisible in the tapestry of digital specimen data. When digitization occurs, it is often done in a piecemeal manner – oftentimes through thematic and regional networks – which results in certain regions and taxonomic groups becoming available online before others. Thus, regions or taxonomic groups with few digital specimens may reflect true low sampling effort, low species abundance, or the lack of digitization of those specimens.

Other biases or errors can be introduced to digital records through typos and misinterpretation of label data, especially if the latter is handwritten from an earlier time. Before analysis, researchers should consider curatorial practices associated with digitization workflows and publishing digital specimen data online. For example, researchers should be clear on the nomenclatures being used and differing practices for translating geographic information or missing information such as dates from specimen labels to digital records. At some herbaria, missing collection dates could be entered as January 1; outside researchers might then wonder why there are so many collections on this day. As another example, of the 48 million preserved specimen records of vascular plants available on GBIF with images at the time of writing this article, only a third have been assigned geographical coordinates. Researchers need to be aware of the varying approaches used to georeference specimens, particularly specimens that lack coordinates, and how this could bias results (Maldonado *et al*., 2015).

### Ethical and social justice considerations

New initiatives to digitize the world's herbaria can raise new and sometimes unexpected ethical and social justice issues. Key ethical and social justice considerations related to working with digital herbarium specimens include equality of access, equality of participation, benefit sharing, and representational accuracy (Miller‐Rushing *et al*., 2022). These ethical and social justice considerations are particularly important in regions of the world dealing with the economic, social, and scientific costs of their colonial legacy (Park *et al*., 2023a). Specimens from these regions are often held outside of their countries of origin, and countries in these regions may have limited funds to support research. Building research teams that include leadership or representation from the involved countries and Indigenous communities can help build capacity in under‐resourced areas. International teams can also help overcome practical barriers, such as language differences and local knowledge needed to access specimens and interpret data (e.g. Lee *et al*., 2022). When building international and cross‐cultural teams, researchers can benefit from research frameworks that are fair and inclusive of cultural differences (Seidler *et al*., 2021; Leliaert *et al*., 2024).

Researchers must also abide by permissions set by herbaria and give appropriate credit. In some cases, appropriate permissions and credits differ among individual specimens or collections within a single herbarium. For example, the Biocultural and Traditional Knowledge Labels projects allow Indigenous communities to add digital labels to specimens, labels that express local and specific conditions for sharing specimens and associated data and for engaging in research (Anderson & Hudson, 2020). The Research Data Alliance also developed a set of CARE principles to guide the management and use of Indigenous data, regardless of where they are held, because mainstream values related to research and data are often inconsistent with Indigenous cultures and collective rights (Carroll *et al*., 2020).

## Future directions

### Continue to expand digitization and analyses in under‐represented areas

Digitization and analysis of specimens collected in parts of the world where few phenology studies have been undertaken – such as the Arctic, which is experiencing the greatest effects of climate change, and the tropics, where species and functional diversity is the greatest – will be an important future direction. Digitization efforts focused on these areas could improve social justice aims by helping people and research communities disadvantaged by colonial legacies *and* helping researchers address critical ecological, evolutionary, and conservation questions (Miller‐Rushing *et al*., 2022; Park *et al*., 2023a). Ideally, these digitization and research efforts will be combined with efforts to increase capacity for previously colonized countries to store and digitize specimens, set research priorities, and analyse data related to specimens that came from those countries (Park *et al*., 2023c).

The continued collection of specimens throughout the world is also called for (Davis, 2023). While a focus on underrepresented regions or taxonomic groups is appropriate, collections in well‐studied floristic regions should also be prioritized for their value for in‐depth investigations of long‐term trends, the responses of individual species, and spatial and temporal patterns of responses.

### Improving the quality and pace of phenology data collection and analysis

Advances in artificial intelligence are rapidly advancing the ability of researchers to characterize and analyse phenology data from digital herbarium specimens (e.g. Davis *et al*., 2020; Pearson *et al*., 2020; Weaver *et al*., 2020; Triki *et al*., 2021; Hussein *et al*., 2022). However, researchers must understand the limitations of this technology and evaluate its appropriateness for automating the task at hand. For example, methods to identify and remove outliers may often be species specific and require knowledge of the biology (e.g. realistic flowering windows) of particular taxonomic groups. Machine learning models used to score phenology in large‐scale analyses of hundreds or thousands of species might be trained on common species or species not matching the full range of possible phenological sequences, and as a result may lead to flawed ways of scoring specimens of unusual or rarer species, leading to erroneous conclusions (Pearson *et al*., 2020; de Lutio *et al*., 2022). For any researcher working with these types of data, it is fundamentally important to know the biology of the species under study.

### Increasing standardization of taxonomy, nomenclature, and sharing analytical results

Incongruencies in taxonomies, nomenclatures, and the recording of location information create obstacles for analysing large data sets of digital herbarium specimens. These types of information are sometimes standardized at the level of individual digitization initiatives. However, efforts to standardize vocabularies, metadata protocols, and data structures across institutions within existing biodiversity data standards, such as Darwin Core (a standardized framework for sharing and organizing biodiversity data), is already removing significant barriers and greatly speeding up the pace of research using herbarium specimens and data linked as part of the extended specimen (Wieczorek *et al*., 2012; Yost *et al*., 2018; Lendemer *et al*., 2020; Pearson *et al*., 2020; Sigwart *et al*., 2025).

### Continuing to address key questions in ecology and evolution

Improvements in the standardization, methods, and regional representation of digitization will open opportunities for future research utilizing digital specimen data. It will be fruitful to continue phenological research in understudied areas, such as the Arctic and tropics, and to explore whether findings from other areas are also found in these regions. For example: Do plastic responses explain more of the variation in flowering time across temperature gradients relative to genetic variation in the tropics and Arctic, as has been found in temperate regions (Ramirez‐Parada *et al*., 2024)? Does hysteranthy serve different functions in the tropics than it does in temperate areas, or does its function vary among taxonomic groups (Buonaiuto *et al*., 2024)? How does urbanization affect flowering phenology outside of temperate areas (Park *et al*., 2023b)? And do we find comparable links between plant traits and phenology in the tropics as has been detected for a large set of herbaceous species monitored in botanical gardens in temperate regions (e.g. Sporbert *et al*., 2022)?

Future research can also address questions, both basic and applied, at scales never previously possible, and for species and phenological events difficult to detect with other types of data (e.g. satellite imagery). For example: How does plant phenology and sensitivity to climate vary across the full ranges of species and within closely related taxonomic groups? How are phenology and plant morphology related across global scales and many different growth forms? How can we better incorporate phenology data from herbarium specimens into forecasts of the impacts of climate change on ecosystems? And can we use specimen‐derived information to better identify plants most vulnerable to climate change?

## Conclusion

The potential for digital herbarium specimens to advance large‐scale phenology research is incredibly exciting. There are many obstacles – the very existence of many herbaria is in doubt due to economic constraints – but there is momentum for researchers to value and use herbaria and other natural history collections to advance ecology, evolution, and climate change biology and to improve society (National Academies of Sciences, Engineering, and Medicine, 2020; Davis, 2023; Johnson *et al*., 2023). We will see new breakthroughs as this work continues, technology advances, and international collaborations grow.

## Competing interests

None declared.

## Author contributions

RBP conceived the article idea together with MWA, NIA, ZAP, CR and AMR. NIA and RBP planned and drafted the manuscript with important writing contributions from MWA, ZAP, CR and AMR. MWA, CR and ZAP produced the figures with contributions from all co‐authors. All authors reviewed the final manuscript.
